# Retention Strength of PMMA/UDMA-Based Crowns Bonded to Dentin: Impact of Different Coupling Agents for Pretreatment

**DOI:** 10.3390/ma8115396

**Published:** 2015-11-06

**Authors:** Bogna Stawarczyk, Simona Teuss, Marlis Eichberger, Malgorzata Roos, Christine Keul

**Affiliations:** 1 Department of Prosthodontics, Dental School, Ludwig-Maximilians University Munich, Goethestrasse 70, Munich 80336, Germany; simona.gilbert@med.uni-muenchen.de (S.T.); marlis.eichberger@med.uni-muenchen.de (M.E.); christine.keul@med.uni-muenchen.de (C.K.); 2 Department of Biostatistics, Epidemiology Biostatistics and Prevention Institute, University of Zurich, Hirschengraben 84, 8001 Zurich, Switzerland; mroos@ifspm.uzh.ch

**Keywords:** CAD/CAM polymer, PMMA, UDMA, retention strength, cementation

## Abstract

Computer aided design/computer aided manufacturing (CAD/CAM) polymers for long-term dental restorations benefit from enhanced mechanical properties. However, the quantification of their bonding properties on teeth is lacking. Therefore, the aim of this study was to determine the retention strength (RS) of differently pretreated new developed polymethylmethacrylate/urethanedimethacrylate-based CAD/CAM polymer bonded on dentin. In summary, 120 human caries-free molars were prepared, and polymeric crowns were milled and pretreated (*n* = 20): visio.link (VL), Scotchbond Universal (SU), Monobond Plus/Heliobond (MH), Margin Bond (MB), Margin Bond mixed with acetone (1:1) (MBA) or not pretreated (CG). Half of the specimens were cemented using Variolink II and the other half with RelyX Ultimate. Specimens were stored for 24 h in distilled water and thermal cycled (5000 ×, 5 °C/55 °C). The retention load was measured and failure types were defined. RS was calculated and analyzed using both two- and one-way ANOVA with a *post-hoc* Scheffé-test, unpaired *t*-test, Kaplan–Meier with Breslow–Gehan test and chi-squared test (*p* < 0.05). Crowns bonded using RelyX Ultimate showed higher RS than those bonded using Variolink II. The pretreatment showed no impact on the RS. However, survival analysis within Variolink II found an impact of pretreatment. The median RS for MH was the lowest and statistically different from MB, MBA and CG. For Variolink II MH had the poorest survival as the estimated cumulative failure function of the debonded crown increased very quickly with increasing TBS. Within the RelyX Ultimate groups, no significant differences were determined. The newly developed CAD/CAM polymer showed the highest bonding properties after cementation using RelyX Ultimate.

## 1. Introduction

In addition to functional necessities, such as protecting the tooth structure from destroying effects and defending the pulp from bacterial colonization and thermal impacts, provisional fixed dental prostheses (FDPs) must also be accommodating from an aesthetic point of view [1]. In the daily clinical routine, traditional options are mainly used for the manufacture of provisional FDPs; however, newer fabrication techniques have become continuously widespread and improved [1]. The traditional techniques involve the chair-side or lab-side making acrylic temporaries on a basis of polymethyl methacrylate (PMMA) and polyethyl methacrylate (PEMA) in the form of a powder/liquid, which requires manually mixing the two components [1]. The drawbacks of the exothermic reaction during intraoral polymerization [2] and shrinkage [3] have been enhanced by the implementation of dimethacrylate-based materials in paste dispensers for the traditional manufacturing technique [1]. The introduction of new processing techniques—such as computer-aided design (CAD)/computer-aided manufacturing (CAM)—has resulted in the further advancement of polymeric materials, used for temporary FDPs. In particular, for high-quality provisional therapy concepts that presuppose a long period of wear, CAD/CAM-produced dental temporaries made of high-density polymers are commonly used clinically [4,5,6].

Compared to manually polymerizing polymers for traditional processing, polymeric CAD/CAM materials exhibit significantly higher physical properties caused by the standardized polymerization under high pressure and temperature [7,8,9]. These include superior mechanical performance [10,11], higher abrasion resistance [12] and inferior discoloration [13]. Particularly for the manual processing of autopolymerizing PMMA, the biophysical properties can be affected by the individual monomer-powder ratio [3].

However, due to the high grade of polymerization, and therefore the scant number of free carbon double bounds [14,15], these polymeric CAD/CAM materials exhibit drawbacks relevant for bonding to natural tooth substrate. Therefore, studies have investigated pretreatment methods to increase the bond strength. Airborne-particle abrasion prior to cementation of polymeric CAD/CAM crowns results in an improvement of bond strength [16]. As a result, the surface of the restoration increases and becomes cleaned simultaneously [16]. In general, sandblasting is more effective for the pretreatment of CAD/CAM polymers than hydrofluoric acid etching or grinding with diamond burs [17,18]. An additional pretreatment of PMMA-based CAD/CAM crowns using coupling agents such as Monobond Plus/Heliobond, visio.link and VP connect showed, after artificial thermal loading, higher bond strength values to resin composite cements than without the coupling agent [19]. For the bond strength of an experimental CAD/CAM nanocomposite to dentin abutments, it was found that both the composite resin cement and coupling agent have an influence on the bond strength [20]. In addition, the curing mode is decisive for the bonding effectiveness of adhesively luted polymeric CAD/CAM restorations to dentin [21].

In general, polymeric CAD/CAM materials consist of a polymeric matrix that is reinforced by inorganic, organic or composite fillers [22]. For a further improvement to the mechanical and optical properties, the trend goes to a higher volume fraction of inorganic fillers into the organic monomer matrix [23]. The appropriate coupling agent for pretreatment of the restoration depends on the composition of the material’s individual components. Due to the increase in inorganic ceramic filler content, development tends toward the use of universal coupling agents. These combine silanes for the pretreatment of ceramic components plus methacrylates for the chemical reaction with carbon double bounds within the organic matrix [21]. An advantage of the incorporation of the silane bi-functional monomer into the coupling agent is the reduced number of working steps [21]. Furthermore, the same bonding agent can be used for the pretreatment of dentine as well as for the pretreatment of the CAD/CAM polymeric dental restoration [21].

Prior research investigated the retention strength of various dental materials to dentin abutments [16,19,20,24,25,26,27]. However, insufficient information is available about the bonding properties of PMMA/UDMA-based CAD/CAM polymeric crowns with additional inorganic filler components to human dentine abutments. Therefore, the following null-hypotheses were tested: (i) pretreatment of CAD/CAM polymeric crowns using different coupling agents; and (ii) different resin composite cements show (a) no impact on the retention strength values to dentin, (b) on the failure types after retention strength measurement, and (c) on the survival analysis of the newly developed CAD/CAM material.

## 2. Material and Methods

This study tested the retention strength of one differently pretreated CAD/CAM-composite based on PMMA and UDMA (XHIPC-CAD/CAM-blank, Xplus3; Echzell, Germany) on dentin. Table 1 describes all materials used. To determine the sample size, a power analysis was calculated using nQuery Advisior (Version 6.0, Statistical Solutions) prior to performing this study. A pilot study (SD = 0.15 MPa) for retention strength of air-abraded CAD/CAM crowns adhesively cemented on dentin abutment was used (same pretreatment, same test method) [16]. A sample size of 10 in each group will behave 99.9% power to detect the difference of 0.44 MPa (increase the values after pretreatment of 20%) using two group *t*-tests with 0.003 Bonferroni corrected two-sided significance level.

**Table 1:** Materials, composition and form of application used in the study.

	Materials	Manufacturer	Lot No.	Compositions
CAD/CAM-blank	XHIPC-CAD/CAM-blank	Xplus3, Echzell, Germany	425120	50%–80%: PMMA
				10%–20%: UDMA, BDDMA, mutli-methacrylate
				app. 15% filler
Pretreatment of dental hard tissue	Total etch	Ivoclar Vivadent, Schaan, Liechtenstein	R29459	37% phosphoric acid
	Syntac Classic	Ivoclar Vivadent, Schaan, Liechtenstein	R46617	Primer: TEGDMA; polyethylene glycol dimethacrylate; maleic acid; acetone in aqueous solution
				Adhesive: polyethylene glycol dimethacrylate; glutaraldehyde in aqueous solution
				Heliobond: Bis-GMA; TEGDMA; stabilizers; catalysts
	Scotchbond Universal *	3M ESPE, Seefeld, Germany	520594	Bis-GMA; HEMA; decamethylene dimethacrylate; silane treated silica; ethanol; water; 2-propenoic acid, 2-methyl-, reaction products with 1,10-decanediol and phosphorous oxide (P_2_O_5_); copolymer of acrylic and itaconic acid; dimethylaminobenzoat(-4); (dimethylamino)ethyl methacrylate; camphorquinone; methyl ethyl ketone
Coupling agent for pretreatment of CAD/CAM crown	visio.link	bredent, Senden, Germany	114784	methyl methacrylate; 2-propenoic acid reaction products with pentaerythritol; diphenyl(2,4,6,-trimethylbenzoyl)-phosphineoxide
	Scotchbond Universal *	3M ESPE, Seefeld, Germany	520594	Bis-GMA; HEMA; decamethylene dimethacrylate; silane treated silica; ethanol; water; 2-propenoic acid, 2-methyl-, reaction products with 1,10-decanediol and phosphorous oxide (P_2_O_5_); copolymer of acrylic and itaconic acid; dimethylaminobenzoat(-4); (dimethylamino)ethyl methacrylate; camphorquinone; methyl ethyl ketone
	Monobond Plus/Heliobond *	Ivoclar Vivadent, Schaan, Liechtenstein	Monobond Plus: R26662;	Monobond Plus: silane methacrylate; phosphoric acid methacrylate; sulphide methacrylate in alcohol solution
			Heliobond: P00865	Heliobond: Bis-GMA; TEGDMA; stabilizers; catalysts
	Margin Bond	Coltène Whaledent, Altstätten, Switzerland	F22965	Bis-GMA; TEGDMA
	Margin Bond 50%	Coltène Whaledent, Altstätten, Switzerland	CK131002	Bis-GMA; TEGDMA; acetone
Resin composite cements	Variolink II	Ivoclar Vivadent, Schaan, Liechtenstein	Base: R46653;	Bis-GMA; TEGDMA, UDMA; barium glass; ytterbium trifluoride; Ba-Al-fluorosilicate glass; spheroid mixed oxide, catalyst, stabilizers, pigments
			Catalyst: LOT P84939	
	RelyX Ultimate	3M ESPE, Seefeld, Germany	509010	glass powder, surface modified with 2-propenoic acid, 2 methyl-3-(trimethoxysilyl)propyl ester and phenyltrimethoxy silane; bulk material; 2-propenoic acid; 2-methyl-, 1,1′-[1-(hydroxymethyl)-1,2-ethanediyl] ester; reaction products with 2-hydroxy-1,3-propanediyl dimethacrylate and phosphorus oxide; TEGDMA; silane treated silica; oxide glass chemicals (non-fibrous); sodium persulfate; TBPIN

* Universal coupling agent containing silanes and methacrylates; TEGDMA: triethylene glycol dimethacrylate; TBPIN: tert. butylperoxy-3,5,5-trimethylhexanoate; BDDMA: buthanediol dimethacrylat; Bis-GMA: bisphenol-A-diglycidylmethacrylate; UDMA: urethane dimethacrylate; PMMA: polymethylmethacrylate; HEMA: hydroxyethyl methacrylate.

### 2.1. Specimen Preparation

For this study, 120 caries-free human molars were collected and kept in 0.5% Chloramine T (Lot. No. 53110, CAS No. 7080-50-4, Sigma-Aldrich, St. Louis, MO, USA) for one week at a room temperature of 23 °C. Subsequently, the teeth were put into distilled water at a temperature of 5 °C for a maximum of six months. The roots of the teeth were embedded in acrylic resin (ScandiQuick; Scan Dia Hans P. Tempelmann GmbH & Co., Hagen, Germany) using a special metal holding device. The device as well as the embedding method is described in detail in previous studies [16,19,20,26,27].

In the next step, all embedded teeth were prepared with a conicity of 10 degrees with a rounded diamond point (grain size: 30 µm) in a hydroelectric turbine (perfecta 900, W & H) fixed in a parallelometer (F4/basic, DeguDent GmbH, Hanau, Germany) to ascertain the parallelism of the tensile force and the dentine walls. The standardized height of the crowns, 3 mm, was obtained by cutting the coronal part in a special cut-off grinding machine (Accutom-50, Struers, Ballerup, Denmark). Subsequently, the rough edges were deburred with a polishing disc (Sof-Lex 1982C/1982 M, 3M ESPE, Seefeld, Germany).

The prepared coronal abutments were scanned for the calculation of the bond area using a lab–side scanner (KaVo Everest Scan, KaVo, Biberach, Germany). Surface tessellation language (STL)-datasets were imported into inspection software (Qualify 12.1.2, Geomagic, Morrisville, NC, USA) and the bond area was calculated for each tooth separately. For the milling process of the CAD/CAM crowns, the teeth were additionally scanned (Ceramill map 400, Amann Girrbach, Koblach, Austria) and polymeric crowns with standardized thickness and external retentions were designed (Ceramill mind, Amann Girrbach). The construction datasets were imported as STL-datasets into a computer system (ZENOTec Cam 3.2 Advanced, Wieland Dental + Technik, Pforzheim, Germany). Crowns were nested into the CAD/CAM blank and milled afterward (I-mes 4020, Wieland Dental + Technik, Pforzheim, Germany).

The inner sides of the resulting crowns were air-abraded using alumina powder with the mean particle size of 50 µm (powder: Orbis Dental Handelsgesellschaft mbH, Münster, Germany; device: Basic Quattro, Renfert, Hilzingen, Germany). Each composite crown was air-abraded for 20 s from a distance of 1 cm and at a 45° angle, and then cleaned in an ultrasonic bath for 5 min.

The 120 abutments with their corresponding crowns were divided into six coupling agent groups (*n* = 20) and treated as follows:

(A) Visio.link (bredent) was applied on the crown surface and polymerized for 90 s.
(B) Scotchbond Universal (3M ESPE) was applied on the crown surface and polymerized for 10 s.
(C) Monobond Plus/Heliobond (Ivoclar Vivadent). Monobond Plus was applied on the crown surface and air-dried for 60 s; Heliobond was applied and polymerized for 10 s.
(D) Margin Bond (Coltène Whaledent) was applied on the crown surface and polymerized for 10 s.
(E) Margin Bond mixed with acetone (1:1) (Coltène Whaledent) was applied on the crown surface and polymerized for 10 s.
(F) No treatment (control group).

For polymerization of visio.link, the polymerization unit Bre.Lux Power Unit (intensity 220 mW/cm^2^; bredent) was used. The remaining coupling agents were polymerized using the LED-unit Elipar S10 (intensity: 1200 mW/cm^2^; 3M ESPE).

Each coupling agent subgroup was again divided into two resin composite cement groups:

(I) Variolink II (Ivoclar Vivadent): the tooth surface was etched (37% phosphoric acid; Total etch) and pretreated according to the manufacturer’s recommendations using the Syntac Classic assortment.
(II) RelyX Ultimate (3M ESPE): the tooth surface was etched (37% phosphoric acid; Total etch) and pretreated according to the manufacturer’s recommendations using Scotchbond Universal.

The dentin pretreatment agents and the resin composite cements were polymerized according to the manufacturer’s instructions (Elipar S10). During the polymerization time, the specimens were loaded with 100 N. After cementation, all specimens were stored in distilled water for 24 h at 37 °C and thermal cycled for 5000 cycles (5 °C/55 °C, dwell time 20 s).

**Figure 1:**
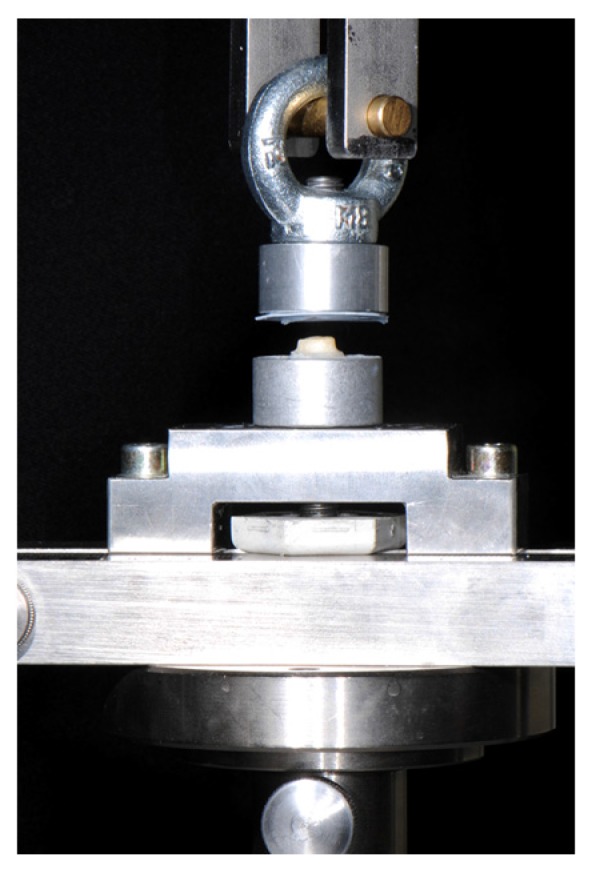
Design of retention strength measurement.

After the artificial aging process, the specimens were released in distilled water for one hour. Subsequently, an upper holding device for the crowns was established as in previous studies [16,19,20,26,27], and the specimens were fixed in a universal testing machine (Zwick 1445, Zwick, Ulm, Germany) and pulled with 1 mm/min until a fracture occurred. The vertical alignment of the specimens during the force application was insured by the toroidal fixation at the upper part of the embedding mold (Figure 1). Retention strength values for specimens that debonded during thermal cycling were set to 0 MPa. After retention strength tests, the failure types were observed by three independent operators using a microscope at 20× magnification (Stemi 2000-C, light source: CL 6000 LED, Zeiss, Oberkochen, Germany). Three failure types were determined:

(i) resin composite cement remains on dentine
(ii) resin composite cement remains on polymeric CAD/CAM crown
(iii) mixed failure of types (i) and (ii)

### 2.2. Statistics

The measured data were analyzed using the Statistical Package for the Social Sciences Version 20 (IBM, SPSS, Statistics, Armonk, NY, USA). Descriptive statistics were computed. The Kolmogorov–Smirnov test was used to test the normality of data distribution. Two- and one-way ANOVA followed by the Scheffé *post hoc* test as well as an unpaired *t*-test were used to determine the significant differences between the pretreatment groups. In addition, retention strength values for specimens, which debonded during thermal cycling, were treated as censored observations. Therefore, the Kaplan–Meier survival estimates and cumulative failure functions (1-Survival) together with the Breslow–Gehan test were computed. A chi-squared test was used to analyze the distribution of failure types in the treatment groups. All results with *p*-values smaller than 5% were considered as statistically significant.

## 3. Results

The descriptive statistics are summarized in Table 2. According to the Kolmogorov–Smirnov test, the values of all groups were normally distributed. Therefore, the data were analyzed using parametric tests. The two-way ANOVA showed a statistically significant impact of tested resin composite cements on the retention strength results (*p* < 0.001). In contrast, the coupling agents (*p* = 0.057) showed no significant impact on the results. However, the interaction between both factors was significant (*p* = 0.010) (Table 3).

**Table 2:** Mean retention strength, standard deviation, and 95% confidence intervals of the resin cements on differently pretreated CAD/CAM resin surfaces and one-way ANOVA followed by Scheffé *post-hoc* between the coupling agents within one resin composite cement. All values are given in MPa. Differing lower case letters indicate significant differences between coupling agents.

Coupling Agents	RelyX Ultimate		Variolink II	
	mean ± SD	95% CI	mean ± SD	95% CI
visio.link	2.08 ± 1.33 ^a^	1.12; 3.03	0.87 ± 0.71 ^a^	0.36; 1.39
Scotchbond Universal	2.13 ± 1.56 ^a^	0.99; 3.25	0.84 ± 0.91 ^a^	0.18; 1.49
Monobond Plus/Heliobond	2.50 ± 1.71 ^a^	1.28; 3.73	0.20 ± 0.03 ^a^	−0.02; 0.42
Margin Bond	2.00 ± 1.26 ^a^	1.09; 2.89	0.48 ± 0.52 ^a^	0.10; 0.85
Margin Bond with acetone (1:1)	3.23 ± 2.70 ^a^	1.29; 5.17	0.44 ± 0.36 ^a^	0.17; 0.70
without	4.49 ± 2.11 ^a^	2.98; 6.01	0.47 ± 0.44 ^a^	−0.14; 0.78

Note: ^a^ Differing lower case letters indicate significant differences between coupling agents.

**Table 3:** Two-way ANOVA results for comparison of retention strength for different coupling agents and resin composite cements and their interaction denoted by “*”.

Source of Variability	Sum of Squares	df	Mean Squares	F	*p*-Value
Constant parameters	324.5	1	324.5	172.7	<0.001
Resin composite cement	143.8	1	143.8	76.5	<0.001
Coupling agents	20.9	5	4.2	2.2	0.057
Resin composite cement *vs.* * coupling agents	29.9	5	6.0	3.2	0.010
Error	202.9	108	1.9	–	–
Total	721.9	120	–	–	–

Crowns bonded using RelyX Ultimate showed, in all coupling agent groups, significantly higher retention strength results than those bonded using Variolink II (*p* < 0.037). The impact of coupling agents was examined using one-way ANOVA for each resin composite cement separately. The coupling agents showed no impact on the retention strength results (*p* > 0.058), regardless of the resin composite cement.

**Figure 2:**
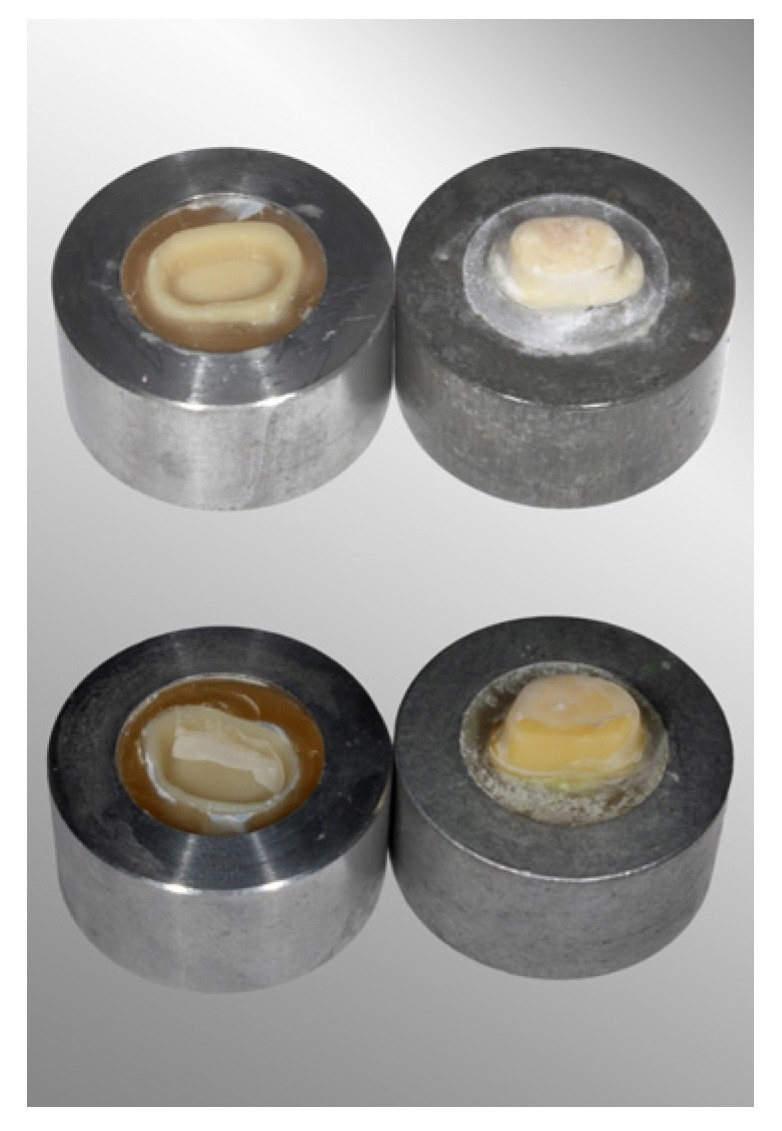
Observed failure types: resin composite cement remaining on dentin (pictured above) and mixed failure type (pictured below) with resin composite cement on dentine and concurrent on CAD/CAM polymer crown.

After the retention strength test, the tested groups showed different failure types that depended on the resin composite cement (*p* < 0.001). Resin composite cement remaining on dentin (Figure 2, pictured above) was observed more frequently for crowns bonded using Variolink II. Mixed failure types (Figure 2, pictured below) were found most frequently in groups bonded using RelyX Ultimate.

**Figure 3:**
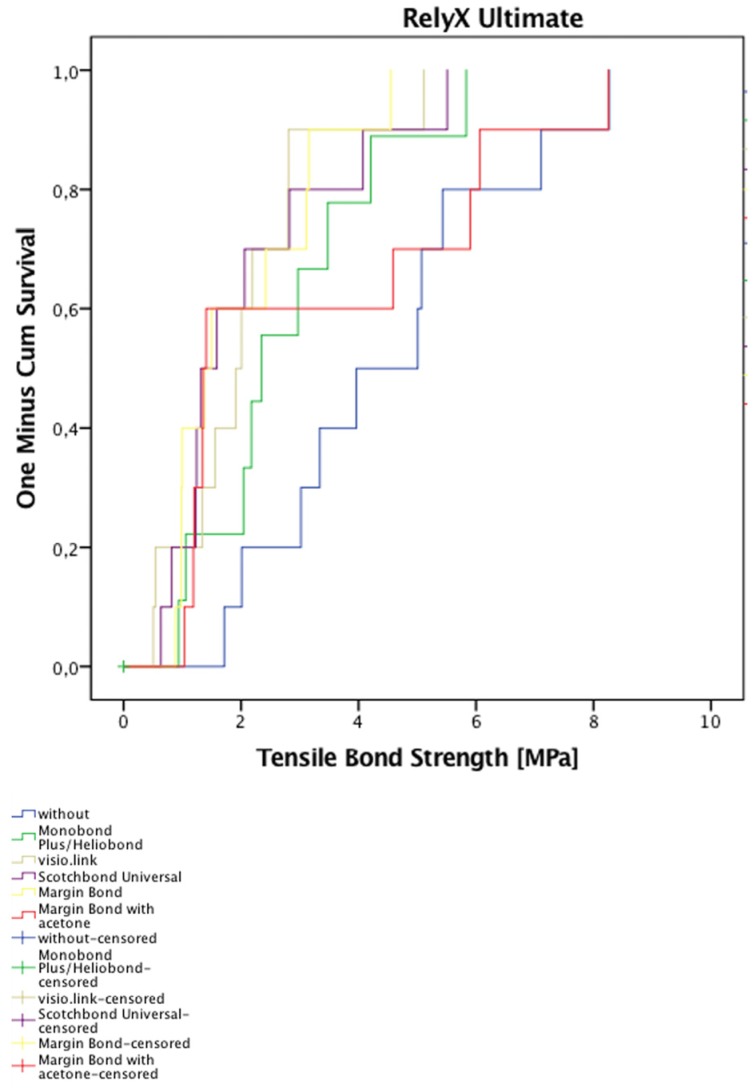
Estimated cumulative failure functions of specimens bonded with RelyX Ultimate.

**Figure 4:**
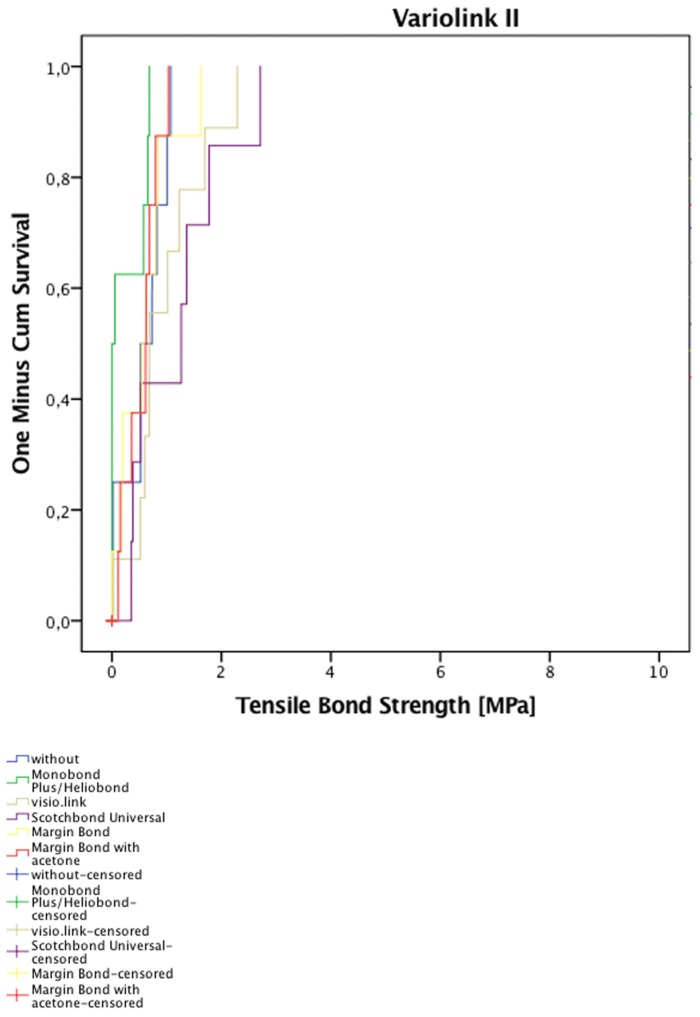
Estimated cumulative failure functions of specimens bonded with Variolink II.

For each separate resin composite cement group, the estimated cumulative failure function of the debonded crown given the retention strength is presented in Figure 3 and Figure 4. These estimates are adjusted for censoring (Table 4). Within the RelyX Ultimate groups, no significant differences were determined according to the Breslow–Gehan test (*p* > 0.083) (Figure 3). In contrast, significant differences were found between the Variolink II groups (*p* = 0.016) (Figure 4). The lowest retention strength in comparison with the remaining groups occurred for the Monobond Plus/Heliobond group. The median retention strength for Monobond Plus/Heliobond (0.004 MPa) was the lowest and statistically different from crowns pretreated with visio.link (0.69 MPa), pretreated with Margin Bond mixed with acetone (0.62 MPa) and crowns without pretreatment (0.52 MPa). Pretreatment with Monobond Plus/Heliobond resulted in the poorest survival because the estimated cumulative function of the debonded crown increased very quickly with increasing retention strength.

**Table 4:** Median survival tensile strength (MPa) and 95% confidence interval of survival in all test groups.

Coupling Agents	RelyX Ultimate Median (95% CI) (MPa)	Variolink II Median (95% CI) (MPa)
visio.link	1.9 (1.21; 2.61)	0.69 (0.66; 0.70)
Scotchbond Universal	1.32 (0.78; 1.86)	1.27 (0.0; 3.18)
Monobond Plus/Heliobond	2.35 (1.85; 2.85)	0.004 (0.0; 0.08)
Margin Bond	1.38 (0.59; 2.15)	0.54 (0.0; 1.14)
Margin Bond with acetone (1:1)	1.35 (1.25; 1.45)	0.62 (0.25; 0.98)
without	3.96 (1.38; 6.55)	0.52 (0.22; 0.82)

## 4. Discussion

The effective cementation of industrially polymerized CAD/CAM polymers is a prerequisite for its use in dentistry as a long-term temporary prosthetic material. Therefore, efficient bond properties should be generated between the different pretreated CAD/CAM polymers and resin composite cements. This study evaluated the bond strength of air-particle abraded PMMA/UDMA-based CAD/CAM polymer to two resin composite cements after using different coupling agents. Air-particle abrasion with alumina powder results in an increase of the inner surface of the dental crown and engenders simultaneously a clean surface for bonding [16]. For the bonding of industrially polymerized PMMA-based crowns, these mechanical retentions are necessary [16]. Therefore, for the current investigation, all CAD/CAM polymer crowns were standardized air-particle abraded using alumina powder with a mean size of 50 µm. In terms of clinical routine, the study included no highly polished polymer specimens. Thus, no statement can be made about the pure chemical bond between the CAD/CAM polymer and coupling agents, or rather, the resin composite cement. For long-term bonding of PMMA/UDMA-based CAD/CAM polymer to tooth surfaces, it could be shown that pretreatment of the CAD/CAM polymer surface with coupling agents had a subordinate role; much more relevant was the choice of resin composite cement. RelyX Ultimate could establish a significant improvement regarding the retention strength and observed failure types. Thus, the hypothesis that the coupling agent has no effect on the retention strength and failure types has to be accepted. The hypothesis that the resin composite cement does not affect the retention strength and failure types has to be rejected.

The present study observed reliable bond strength of RelyX Ultimate to CAD/CAM polymers. This observation was supported by the analysis of failure types; namely all groups cemented with RelyX Ultimate showed predominantly mixed failures, while groups cemented with Variolink II resulted in mainly adhesive failures with resin composite cement remaining on dentine. This emphasizes the proposition that the bond strength of RelyX Ultimate to the polymer material is stronger than the bond strength of Variolink II. In general, prior literature observed that after application of coupling agents, there was an increase of the bond strength values of resin composite cements to CAD/CAM materials on a PMMA or composite basis [14,15]. Contrary to this, in view of the present results, it can be stated that the coupling agent had no impact on the retention strength values. More negatively, the combination Monobond Plus/Heliobond showed the lowest survival rate within with Variolink II cemented polymer crowns. Thus, the hypothesis that the coupling agent and the resin composite cement have no effect on the survival rate has to be rejected.

Another method for modification of the CAD/CAM polymer surface is the chemical breaking of the carbon double bonds using acetone in a solution. A slight dissolving of the polymeric matrix on the inner side of the crown can be presumed by the solvent capacity of the acetone. However, the supplementary addition of 50% acetone to the Margin Bond coupling agent resulted in no increase of the retention strength for both investigated resin composite cements. Visio.link contains MMA and dimethacrylate and resulted in prior investigations in a positive effect on the bonding properties between resin composite cements and industrially polymerized CAD/CAM polymers [14,15]. The results of the present study cannot confirm these findings.

During the planning of this study the mean effect of 0.44 MPa was assumed. In the present study, effects as large as 2.49 MPa for RelyX Ultimate and 0.67 MPa for Variolink could be observed. The assumed standard deviation of 0.15 MPa could not be confirmed in our study. The observed pooled standard deviations were 1.85 MPa and 0.57 MPa for measurements within RelyX Ultimate and Variolink II resin composite cements, respectively. The increased spread of measurements could be partly explained by the debonding of specimens during thermal cycling. For the planned statistical analysis, these measurements were coded as 0 MPa. The *post-hoc* power analysis revealed that due to increased spread, the actual power was 37% and 25% for RelyX Ultimate and Variolink II, respectively. Given the observed effects and standard deviations, at least 19 (24) specimens for each coupling agent within RelyX Ultimate (Variolink II) would be necessary to achieve the power of 80%. This is a clear limitation of this study.

In previous studies, retention strength to dentin abutment was observed for standard gold crowns: 0.60–2.36 MPa [24]; adhesively bonded zirconia crowns: 2.6–14.1 MPa [25,26]; air-abraded and adhesively bonded PMMA-based CAD/CAM crowns: 1.9–2.6 MPa [16]; PMMA-based CAD/CAM crowns after pretreatment using different coupling agents: 0.69–2.3 MPa [19]; CAD/CAM nanocomposite crowns after pretreatment using coupling agents: 0.16–4.06 MPa [20]; and adhesively bonded PEEK-based crowns: 0.34–2.97 MPa [27]. In this study, the measured tensile bond strength (0.2–4.49 MPa) of PMMA/UDMA-based CAD/CAM crowns to resin composite cements was lower compared to zirconia crowns. However, the values in this study were comparable to retention strength values of gold, PMMA-, PEEK-based and composite CAD/CAM crowns.

The present study used the pull-off test for the determination of the bond strength between adhesively cemented CAD/CAM polymer and human tooth substrate. This method nearly simulates the clinical situation of CAD/CAM polymer crowns in the oral cavity. However, for the present test design not only tensile stresses were measured, but rather a mix of tensile stresses at the occlusal area and shear stresses at the axial walls. All extracted human teeth were stored after the extraction according to the ISO 11405 guideline [28]. Regarding the methodology, extracted human teeth were manually prepared with diamond preparation burs under a continuous water supply using a parallelometer. However, as consequence, there may have been unstandardized, operator dependent contact pressure of the dental drill and water supply. In addition, as the study included human material, the individual variation of the substrates had to be considered. In contrast, studies on the basis of general geometric shaped bodies can produce more standardized specimens, with less individual variability. Due to the use of non-vital tooth tissue, no intrapulpal pressure could be simulated, which might have influenced the bonding *in vivo*. These factors have to be seen as limitations of the present study because the results may at least vary for vital teeth.

Commercial analyzing software (Geomagic Qualify 12.1.2) was used for the determination of the bonding area, which allowed the calculation on basis of the STL surface datasets. Compared with previously published data [24,25], this calculation method presents more precise results.

Thus, for these reasons, it is important to acknowledge that the conditions of the present investigation differ from *in vivo* situations and that a general comparability with other studies is difficult. Nevertheless, as this was a comparative investigation, the findings of this study allow at least the detection of pretreatment and resin composite cement differences when using one CAD/CAM polymer material under standardized conditions.

## 5. Conclusions

Within the limitations of this study, the following conclusions can be drawn:

1. The tested universal coupling agents showed no impact on the retention strength results.
2. Pretreatment with Margin Bond compared to Margin Bond mixed with acetone showed no impact on the retention strength results.
3. Crowns cemented using RelyX Ultimate showed higher retention strength than those polymerized with Variolink II.
4. Mixed failure types were found most frequently in groups bonded using RelyX Ultimate. In contrast, composite resin cement remaining on dentin within crowns bonded using Variolink II.
5. Within Variolink II groups, crowns pretreated with Monobond Plus/Heliobond showed a lower estimated cumulative function of the debonded crown than the remaining pretreatment groups.
